# Genotypic and Phenotypic Heterogeneity in *Alicyclobacillus acidoterrestris*: A Contribution to Species Characterization

**DOI:** 10.1371/journal.pone.0141228

**Published:** 2015-10-20

**Authors:** Antonio Bevilacqua, Monica Mischitelli, Valeria Pietropaolo, Emanuela Ciuffreda, Milena Sinigaglia, Maria Rosaria Corbo

**Affiliations:** 1 Department of the Science of Agriculture, Food and Environment (SAFE), University of Foggia, Via Napoli, 25, 71122, Foggia, Italy; 2 Department of Public Health and Infectious Diseases, Sapienza University of Rome, P.le Aldo Moro, 5, 00185, Rome, Italy; University of Campinas, BRAZIL

## Abstract

*Alicyclobacillus acidoterrestris* is the main cause of most spoilage problems in fruit juices and acidic products. Since soil borne species often contaminate fruit juices and do not need strict extreme requirements for survival, it is a great concern to investigate whether and how soil species could evolve from their ecological niches in microbial community to new environments as fruit juices. In this study, 23 isolates of thermo-acidophilic, spore-forming bacteria from soil were characterized by cultural and molecular methods. In addition, 2 strains isolated from a spoilage incident in pear juice were typed. Strains phenotyping showed that they could be grouped into 3 different clusters, and some isolates showed identical or quite similar patterns. Analyzing pH and temperature ranges for growth, the majority of strains were able to grow at values described for many species of *Alicyclobacillus*. Qualitative utilization of lysine, arginine and indole production from tryptophan revealed, for the first time, deamination of lysine and decarboxylation of arginine. Resistance to 5% NaCl as well as the ability to hydrolyze starch and gelatin, nitrate reduction, catalase and oxidase activities confirmed literature evidences. Examining of 16S rRNA, showed that isolates were divided into three blocks represented by effectively soil species and strains that are moving from soil to other possible growing source characterized by parameters that could strongly influence bacterial survival. RAPD PCR technique evidenced a great variability in banding patterns and, although it was not possible to obtain genotypically well-distinguished groups, it was feasible to appreciate genetic similarity between some strains. In conclusion, the investigation of a microbial community entails a combination of metagenomic and classic culture-dependent approaches to expand our knowledge about *Alicyclobacillus* and to look for new subspecies.

## Introduction

In 1987, Deinhard and coworkers proposed that the species of *Bacillus* mainly isolated from soil and characterized by acid loving behavior would be called *acidoterrestris* [1]. In 1992, a study carried out by Wisotzkey and colleagues focused on the proper taxonomic placement of *B*. *acidoterrestris* and its closely related species, i.e. *B*. *acidocaldarius* and *B*. *cycloheptanicus*. 16S rRNA comparative sequence and secondary structures were investigated and the new genus *Alicyclobacillus* was recommended [2]. *Alicyclobacillus* genus grouped Gram positive, thermo-acidophilic, rod-shaped, non-pathogenic and spore-forming microorganisms with unusual *ω*-alicyclic fatty acids in their cell membrane. Moreover they can grow in highly acidic environments (2.5–6.0 pH) and at temperatures of 25–60°C [3,4]. *Alicyclobacillus acidoterrestris* is composed of bacilli usually isolated from soil (primary source), plants, spoiled juices, tea and equipments (secondary sources) [5]. The main characteristic of these species is the ability to spoil juices and acidic products by producing guaiacol and other halophenols [2; 6–8]. Cerny et al. [9] first recognized thermophilic bacilli, later called *A*. *acidoterrestris*, as the leading microorganism of apple juice spoilage in Germany in 1982. Since then, thermo-acidophilic bacilli were implicated in juice spoilage incidents in several countries [10–11]. Spoilage occurred in early spring or summer and most commonly in apple juice, though reports of other beverages and tomato juice were also described [5; 12–13]. Spoilage in consumer packaged products has been widely described as “Smoky bacon”, “Hammy” or even “Antiseptic”. Companies often did not realize a spoilage incident until they received consumer complaints. Resistance to pasteurization temperatures, low pH and the ability to produce off flavors, led researchers to acknowledge *Alicyclobacillus* spp. as an important target in the quality control of acidic beverages [14–16]. It is commonly believed that *A*. *acidoterrestris* is the main cause of most spoilage problems; however *A*. *acidiphilus* and *A*. *herbarius* can also produce guaiacol [17]. Moreover, since soil borne species often contaminate fruit juices and do not need strict extreme requirements of acidity and high temperature for survival, it is a great concern to investigate whether and how soil species could evolve from their ecological niches in microbial community to new environments as fruit juices. Several questions can be posed: soil species could acquire specific phenotypic traits in relation to available nutrients? Phenotypic diversity reflects genotypic variations? Among soil species could be present a “quasi-species” that could represent an intermediate between different evolutionary states? To address these issues, 23 isolates of thermo-acidophilic, spore-forming bacteria from soil and 2 strains from spoiled pear juice were characterized at biochemical and molecular levels to appreciate genomic and phenotypic differences between environment and juice bacteria.

## Materials and Methods

### Reference Cultures and Isolation of Gram positive spore forming bacteria

25 strains of thermo-acidophilic, spore-forming bacteria were used throughout this study. Strains belong to a private collection of the Department of the Science of Agriculture, Food and Environment (SAFE) (Foggia University). 23 strains were randomly selected from soil of Foggia (peach orchard, cornfield, ploughed field, garden suburb) and named with capital letter C and 2 strains isolated from a spoilage incident in pear juice. These ones were named CB-1 and CB-2. A partial sequence (1406 bp) corresponding to the 16S ribosomal RNA gene of CB-1 strain was submitted to GenBank database and the accession number assigned was KP144333.

10 g of soil were diluted with 90 ml of sterile saline solution (0.9% NaCl) and homogenized for 1 min. Thereafter, this suspension was heat-treated at 80°C for 10 min, serially diluted in saline solution and plated on Malt Extract Agar (MEA) (Oxoid, Milan, Italy), acidified to pH 4.5 through a sterile solution of citric acid (1:1,w/w); the plates were incubated at 45±1°C for 3–5 days.

Spoiled pear juice was serially diluted in saline solution, heat treated and analyzed as reported above. From each plates, Gram-positive and spore-forming strains were selected. The isolated strains were stored on acidified MEA slants at 4°C.

### Spore production

Spores were produced on acidified MEA, incubated at 45±1°C for 7 days until approximately 70–80% of cells sporulated. Spores were removed by a mild agitation of plates using a glass spreader after adding 5 ml of distilled water; spore suspension was centrifuged at 1,000 g for 10 min, after which the supernatant was discarded and the pellet resuspended. Spores were cleaned by washing the pellets with sterile distilled water, followed by centrifugation; then, spore suspension was heated at 80°C for 10 min to eliminate vegetative cells and stored at 4°C. Spore number was assessed through the spread plate count on acidified MEA, incubated at 45±1°C for 2 days and reported as log cfu/ml.

### Phenotyping

The phenotyping of the strains was based upon the following test:

- Gram staining.
- Catalase and oxidase test.
- Oxido-fermentative metabolism in Hugh-Leifson broth (Biolife, Milan) acidified to pH 5.5.
- Growth in anaerobiosis in Malt Extract broth, incubated for 7 days, and covered with paraffin oil.
- Hydrolysis of starch on PCA (Plate Count Agar, Oxoid), added with starch (10 g/l) (C. Erba, Milan), and acidified to pH 5.5.
- Voges-Proskauer reaction, as reported by Tiecco [18].
- Reduction of nitrate in Nitrate broth (bacteriological peptone, 8.6 g/l-Oxoid; NaCl, 6.4 g/l-J.T. Baker; KNO_3_, 1.5 g/l-J.T. Baker) acidified to pH 4.5.
- Decarboxylation of lysine on Lysine Iron Agar (Oxoid), acidified to pH 4.5.
- Deamination of arginine in the broth of Abd-El Malek (tryptone, 5.0 g/l-Oxoid; yeast extract, 2.5 g/l-Oxoid; glucose, 0.5 g/l-J.T. Baker; K_2_HPO_4_, 2.0 g/l-J.T. Baker; arginine, 3.0 g/l-Sigma Aldrich).
- Production of indole from tryptophane.
- Hydrolysis of gelatine in Nutrient Gelatine (Oxoid), acidified to pH 5.5.
- Hydrolysis of esculin in acidified Malt Extract broth, supplemented with esculin (2.0 g/l) (Sigma-Aldrich) and Fe-ammonium citrate (1 g/l) (J.T. Baker).
- Urea hydrolysis in Urea broth (Oxoid), acidified to pH 4.5.
- Production of H_2_S on SIM medium (Oxoid).
- Growth in acidified Malt Extract broth, supplemented with 0.02% Na-azide (Sigma-Aldrich).

All the media, when required, were acidified through a solution of citric acid (1:1, J.T. Baker, Milan, Italy) and e incubated at 45±1°C

### Effect of pH, NaCl, temperature

Growth profile of alicyclobacilli was assessed as follows:

- acidified Malt Extract broth, supplemented with NaCl (2, 5, 7, 8%), incubated at 45±1°C;
- Malt Extract broth buffered to 2.5, 3.0, 3.5, 4.0, 4.5, 5.0, 5.5, 6.0, 6.5, and 7.0 (±0.05) and incubated at 45±1°C;
- acidified Malt Extract broth incubated at 25, 30, 35, 40, 45, 50, 55, 60, 65, and 70°C (±1°C).

The samples were inoculated to 6 log cfu/ml with either cells or spores and incubated for 7 days: growth was assessed every day by measuring absorbance at 420 nm through a spectrophotometer UV-VIS DU 640 Beckman (Fullerton, CA).

Results from phenotyping were converted into quali-quantitative codes (0, no growth or no enzymatic activity; 1, growth or positive enzymatic activity) and used as input data to run a cluster analysis through the add-in component of Excel XLSTAT (Addinsoft, Paris, France).

### Sample preparation and DNA extraction

Bacteria were taken at 24 h and 48 h and suspended in 1 ml of distilled water until 1 Mac Farland (about 10^6^ cfu) was reached.

DNA extraction was carried out using Norgen’s Bacterial Genomic DNA Isolation Kit (Norgen Biotek Corp.3430 Thorold Ontario, Canada) according to the manufacturer’s instructions. Purification is based on spin column chromatography as the separation matrix. Columns bind DNA under high salt concentrations and release the bound DNA under low salt and slightly alkali conditions.

Extracted DNA was measured at 260 nm and at 280 nm to verify the presence of cellular proteinaceous components. Maximum yields of clean DNA was recovered from 24 h samples; hence, these specimens were used for further analyses.

### Strain identification

In order to provide genus and species identification for isolates, biochemical profiles, 16S ribosomal RNA gene (16S rDNA) amplification and sequencing and Random-Amplified-Polymorphic-DNA (RAPD) were performed.

#### Biochemical profiles

The biochemical profile of the strains was assessed on pure cultures through the miniaturized system API 50 CH, using the suspension medium 50 CHB (Biomeriux, Marcy L’Etoile, France). The API test was incubated at 45 (±1) ^°^C for 48 h.

#### 16S ribosomal RNA typing

16S rDNA was obtained by means of a specific Polymerase Chain Reaction (PCR) using two universal primers (Fw: 5’-AGAGTTTGATCCTGGCTCA-3’, positions 8 to 26; Rw: 5’-CGGCTACCTTGTTACGGAC-3’, positions 1511 to 1493, in the *Escherichia coli* numbering system) [2]. Reaction mixture was prepared in a total volume of 25 μl and it was composed of 200 ng of template, 0.1 pmol/μl of each primer, 200 μM of each deoxynucleoside triphosphate, 2 U/μl of Taq DNA polymerase (ThermoScientific DreamTaq Green DNA Polymerase) 2.5 μl of 10X PCR buffer supplied with the enzyme and 2 mM of MgCl_2_.

Amplification was carried out in GeneAmp PCR System 9600 (Perkin-Elmer Cetus, Emeryville, CA) and consisted of initial denaturation at 94°C for 2 min followed by 35 cycles divided in denaturation step for 20 s at 95°C, primer annealing for 40 s at 55°C and elongation for 2 min at 72°C. A final elongation step was added at 72°C for 5 min.

All assays included positive (*A*. *acidoterrestris* DSM 3922^T^ and *A*. *acidoterrestris* DSM 2498) and negative (all the PCR components except the template) controls to exclude false-positive and false-negative results. Reference strains are personal property of the Department SAFE.

Amplicons were evaluated with 1.0% agarose gel electrophoresis in TBE buffer using the DNA molecular mass marker O’GeneRuler 1 kb DNA Ladder (Thermo Fisher Scientific Inc. MA USA). Proper size products (1500 bp) were purified using an appropriate commercial kit (QIAquick PCR purification kit) and sequenced by a service facility (BioFab research s.r.l., Rome, Italy) through Sanger dideoxy sequencing.

Basic Local Alignment Search Tool analyzed acquired sequences at NCBI website whereas alignments were performed with ClustalW2 at the EMBL-EBI website using default parameters.

Evolutionary analysis, based on Neighbor-Joining method, was conducted in MEGA software version 6.0. Adopted reference strains were *A*. *acidoterrestris* DSM 3922^T^, *A*. *acidoterrestris* DSM2498 and *A*. *acidoterrestris* 49025 whose sequences were taken from GenBank, (accession number: AB042057).

*B*. *subtilis* IAM 12118 ^T^ was used as an outgroup. Its 16S ribosomal RNA sequence was obtained at GenBank, (accession number: AB042061.1).

#### Random-Amplified-Polymorphic-DNA

Random Amplified Polymorphic DNA (RAPD) was done using three primers, Ba-l0 (5'-AACGCGCAAC-3), F-64 (5'-GCCGCGCCAGTA-3') and F-61, (5'-CCTGTGATGGGC-3’) [19]. DNA amplification was carried out in a total volume of 10 μl containing 0.32 pmol/μl of primer, 200 μM of each deoxynucleoside triphosphate, 2 U/μl of Taq DNA polymerase (ThermoScientific DreamTaq Green DNA Polymerase), 1 μl of 10X PCR buffer supplied with the enzyme and finally 3 mM of MgCl_2_.

Template was tested at the final concentration of 30 ng/μl, 20 ng/μl and 15 ng/μl. Each sample was tested in triplicate in order to obtain repeatable results and to choose the better DNA concentration suitable with amplification.

All assays included positive (*A*. *acidoterrestris* DSM 3922^T^ and *A*. *acidoterrestris* DSM 2498) and negative (all the PCR components except the template) controls to exclude false-positive and false-negative results.

The thermal cycling was done in a GeneAmp PCR System 9600 (Perkin-Elmer Cetus, Emeryville, CA) according to the following program: initial denaturation at 94°for 2 min followed by 50 cycles of 94°C for 4 s, 45°C for 8 s, 72°C for 40 s and a final extension at 72°C for 3 min. Electrophoresis for PCR products was made on 2.0% agarose gel in TBE buffer with the DNA molecular mass marker O’GeneRuler 100 bp DNA Ladder (Thermo Fisher Scientific Inc. MA USA). DNA profile of each specimen was visualized under UV light (254 nm), captured using photography apparatus (Kodak DC 290) and analysed with KODAK 1D LE 3.6 software.

## Results and Discussion

In order to understand whether *A*. *acidoterrestris* could adapt in different ecological niches, 23 isolates of thermo-acidophilic, spore-forming bacteria taken from ground were characterized by using cultural (morphological, physiological and biochemical) and molecular (16S rDNA and RAPD) methods. In addition, 2 strains isolated from a spoilage incident in pear juice were typed to appreciate genomic differences between soil and juice bacteria.

### Phenotyping

**Fig 1:**
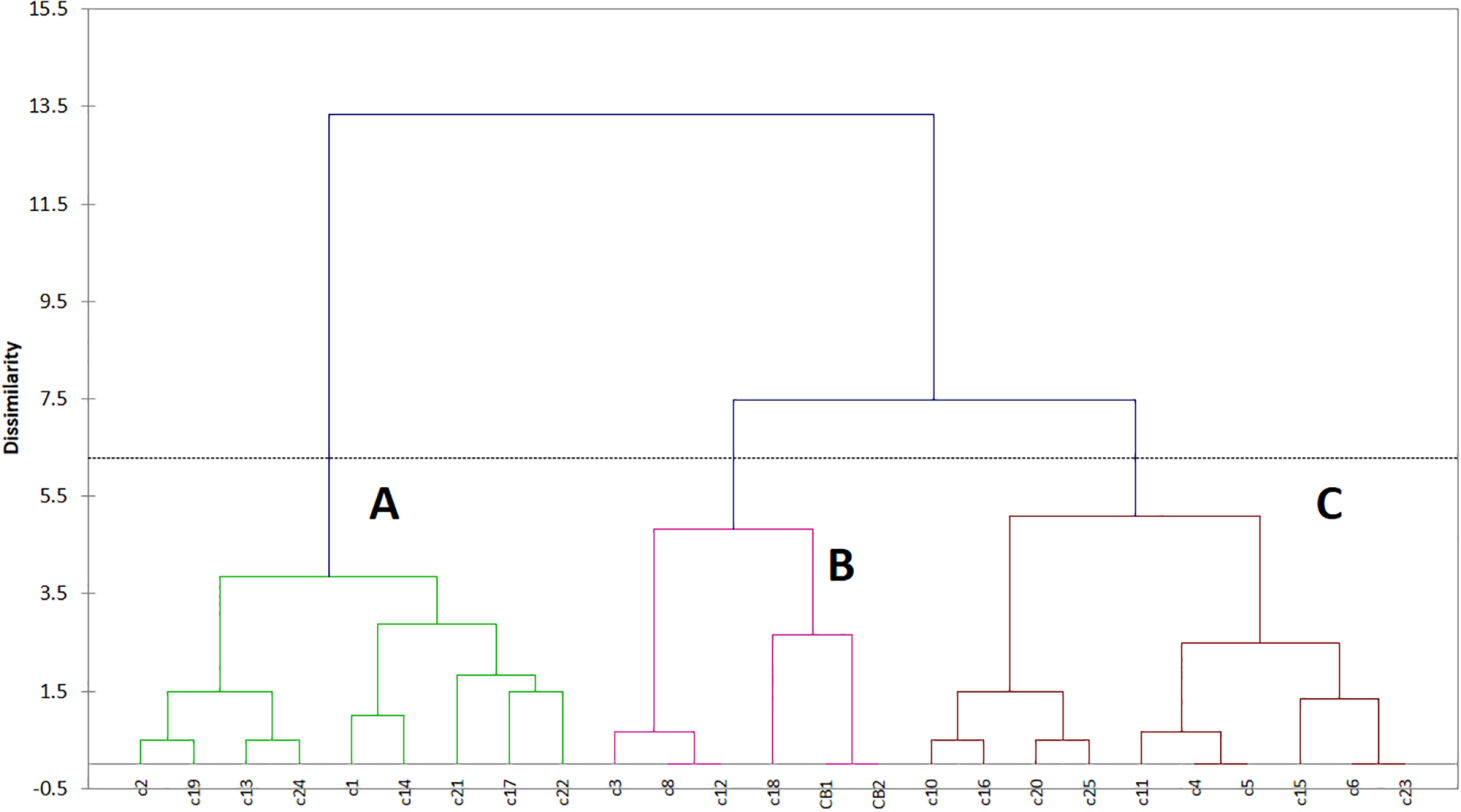
Clustering of isolated *A*. *acidoterrestris* strains considering some biochemical traits.

Phenotyping was based upon the assessment of growth at different temperatures, NaCl concentrations, and pH values, as well as on the evaluation of some biochemical traits (amino acid utilization, production of some secondary metabolites etc…). The results from these assays (growth/no growth; positive/negative) were converted into qualitative binary codes (0 and 1) and used as input values to run a Cluster Analysis. Fig 1 shows the results of this multivariate approach. Strains could be grouped into 3 different clusters (A, B and C), containing respectively 9, 6, and 10 isolates. Some strains showed identical phenotypic patterns (C8 vs C12; CB-1 vs CB-2; C4 vs C5; C6 vs C23) or quite similar (C2 vs c19; C13 vs C24; C10 vs C16; C20 vs C25); the strains from spoiled pear juice (CB-1 and CB-2) were in the cluster B, with some isolates from soil (C3, C8, C12, C18).

Tables 1 and 2 show pH and temperature ranges for growth; many strains were able to grow at pH 2.5, with some exceptions to this generalized statement (strains CB-1, and CB-2 from juices and C1, C3, C8, and C12 from soil). The maximal pH was from 5.5 to 6.5. Concerning temperature, the minimal value was quite variable from 25°C (many isolates from soil) to 35°C (CB-1, CB-2, C18, and C21); a strong variability was also found for the maximal temperature (from 55°C for CB-1 and CB-2 to 70°C for the strains C13, C19, C24, and C25).

**Table 1:** pH growth profile of *A*. *acidoterrestris* strains (the error of pH was ±0.05).

Strains	pH range (min-max)
C2, C4, C5, C6, C11, C19, C21, C22, C23, C24	2.5–6.5
C13, C20, C25	2.5–6.0
C10, C14, C15, C16, C17, C18	2.5–5.5
CB-1, CB-2	3.0–6.5
C1, C3, C8, C12	3.0–5.5

**Table 2:** Temperature profile of *A*. *acidoterrestris* strains (the error of T was ±1°C).

Strains	Growth temperature (°C) (min-max)
C13, C19, C24, C25	25–70
C1, C2, C10, C14, C20	25–65
C3, C4, C5, C6, C8, C11, C12, C15, C16, C22, C23	25–55
C17	30–55
C18, C21	35–60
CB1, CB2	35–55

These ranges of pH and temperature for growth have been found and described for many species of *Alicyclobacillus*, isolated from soil and acidic beverages (*A*. *pomorum*, *A*. *herbarius*, *A*. *contaminans*, *A*. *sacchari*, *A*. *acidoterrestris*, *A*. *sendaiensis*) [2; 17; 20–22]. The description of the genus *Alicyclobacillus* was greatly modified from the 1^st^ report by Wisotzkey et al. [2], due to the recovery of many species and subspecies from different environments and sources. Nowadays, the genus includes acidophilic species and thermophiles, with growth range of 35–60°C and pH 3–6, but some strains can grow at room temperature (20–30°C) and at pH around neutrality (6.5–7.0) [17].

**Fig 2:**
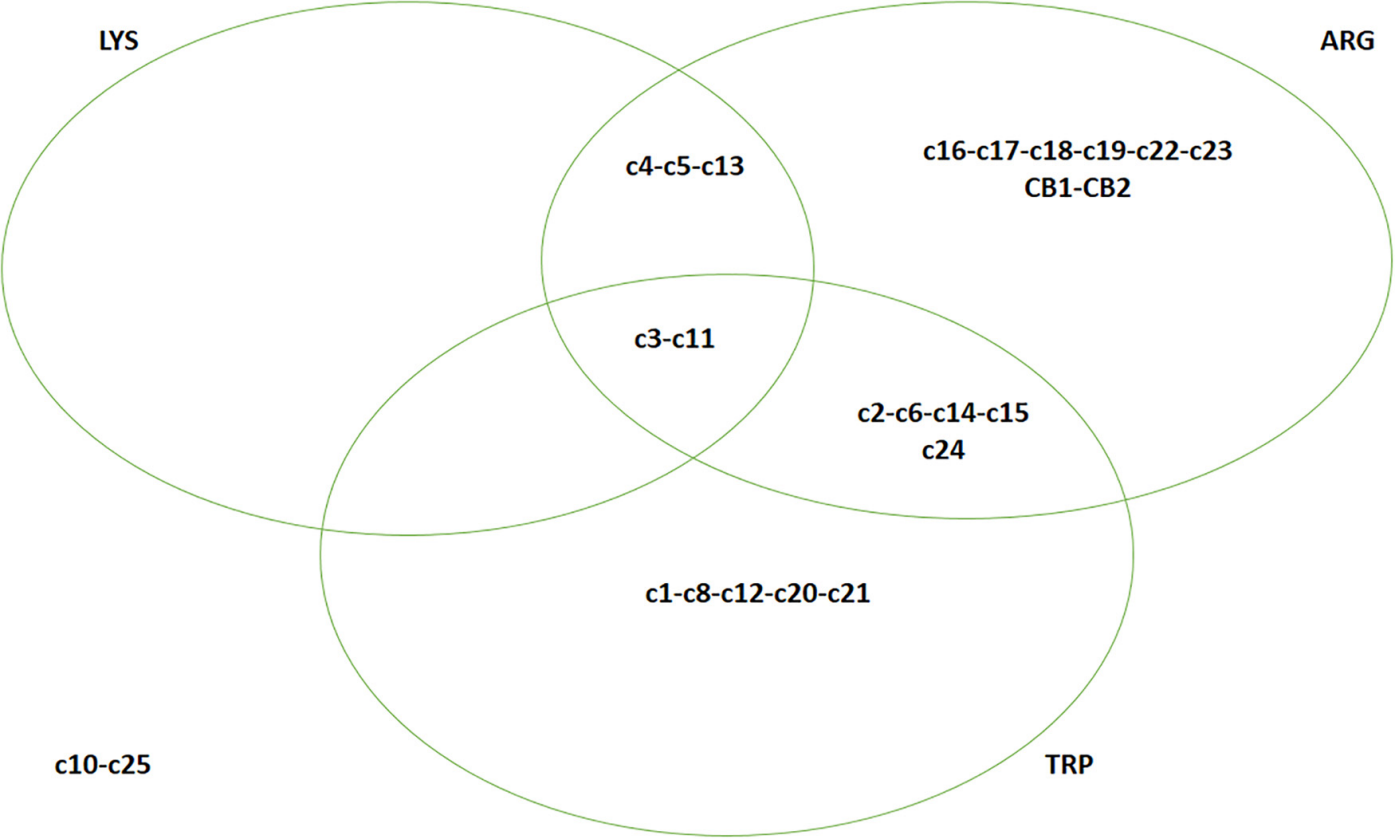
Eulero-Venn diagram for amino-acid utilization. ARG, deamination of arginine; LYS, decarboxylation of lysine; TRP, production of indole.

Some interesting results were recovered from the assays on the qualitative utilization of lysine, arginine and indole production from tryptophan (Fig 2). Two strains used all the amino acids (C3 and C11) and many others used 2 out of 3 compounds (arginine and tryptophan for 5 strains and lysine/arginine for 3 strains); finally, 14 isolates used either arginine or tryptophan, but not lysine alone. Indole production was used as a taxonomic test for the characterization and classification of many *Alicyclobacillus* spp. and this trait is variable within the genus, thus confirming the results of this paper [20–27]. This is the 1^st^ report on the deamination of lysine and decarboxylation of arginine by alicyclobacilli, thus further investigations are required to elucidate the biochemical mechanisms underlying these pathways.

Alicyclobacilli growth was found in Malt Extract broth with 5% salt, except for the isolates C6, C15, C23, CB-1 and CB-2; moreover, some strains could also grow in presence of 8% NaCl (C1, C13, C14, C17, C21, C22, C24). Concerning the other metabolic traits, only one strain could hydrolyze starch and gelatin whilst catalase and oxidase tests were strain-dependent. Resistance to 5% NaCl is a variable trait inside the genus *Alicyclobacillus*, as well as the ability to hydrolyze starch, and gelatin, nitrate reduction, catalase and oxidase activities [22], thus the results of this paper confirm these literature evidences, although they also suggested a higher resistance to salt of our strains. Finally, all the strains could grow in Malt Extract broth covered with paraffin oil; the ability to grow in micro-aerophilic environments was first reported by Goto et al. [23], although *A*. *acidoterrestris* was described as a strict aerobic species.

### Genotyping

16S RNA typing revealed that the isolates belonged to *Alicyclobacillus* genus. The cut-off point for the identification of the genus is a 95% homology [28]; however due to the peculiarity of *Alicyclobacillus* spp., novel species and some strains of the classical species show a lower homology (for example 92.3% *A*. *contaminans* and *A*. *fastidiosus*; 91.3% *A*. *herbarius*; 90.3% *Alicyclobacillus tengchnogensis*; 92.1% for *A*. *tolerans*) [20, 22, 29, 30]. Thus, the strain C2 could be attributed to *Alicyclobacillus*, whilst the results for the strain C4 should be confirmed by further investigations, due to the low homology (89%) and the difficulty to obtain pure DNA. At species level, many isolates (C3, C5, C6, C8, C11, C12, C13, C14, C15, C16, C17, C18, C19, C20, C21, C22, C23, C24, C25, CB-1, CB-2) possessed high homology values with the type strains *A*. *acidoterrestris* DSM3922^T^ and *A*. *acidoterrestris* 49025 (Table 3) The strains C1, C2, C10 showed a 93–95% homology; this value is lower than the international recognized cut-off point for species identification. However, the alignment was performed on both 16S ribosomal and NCBI nucleotide databases and the identity score was >200. For the isolates C2 and C5 the lower homology could be due to the length of the sequences (675 for C2 and 698 for C5). A sequence of 600–700 bp could be referred as unbelievable for the identification; however, DNA extraction from alicyclobacilli could be a great challenge. In fact, Groenwald et al. [31] identified these microorganisms with sequences of 800 bp or lower and highlighted the importance of achieving pure and repeatable sequence in the region of genus identification, with a focus on species diversity. For these reasons and for the results reported in the following section, the strains C1, C2 and C5 were attributed to *A*. *acidoterrestris* species.

**Table 3:** Identity percentage between 16S sequences obtained from studied strains and 16S sequences filed at NCBI website. The strain CB-1 was referred as “γ4” in Bevilacqua and Corbo [5].

SAMPLE	REFERENCE STRAIN(NCBI 16S RIBOSOMAL DATABASE)	IDENTITY	STRAIN(NCBI NUCLEOTIDE DATABASE)	IDENTITY
C1	DSM 3922	935/982 (95%)	FB40 (KF880723)source:debris from factory floor	935/982 (95%)
C2	DSM 3922	628/675 (93%)	FB40 (KF880723)source: debris from factory floor	628/675 (93%)
C3	DSM 3922	695/698 (99%)	FB40 (KF880723)source: debris from factory floor	695/698 (99%)
C4*	DSM 3922	602/674 (89%)	FB40 (KF880723)source: debris from factory floor	602/674 (89%)
C5	ATCC 49025	680/698 (97%)	UFRRJAT1 (KC783431.1)source:orange juice	686/698 (98%)
C6	DSM 3922	890/898 (99%)	C-ZJB-12-31 (KC354628)source: fruit from the orchard in Bairui kiwi fruit Experimental Base	895/898 (99%)
C8	ATCC 49025	1400/1407 (99%)	XC-6 (KJ158157)source: maize juice	1396/1401 (99%)
C10	DSM 3922	950/995 (95%)	C-ZJB-12-36 (KC354633)source: fruit from the orchard in Bairui kiwi fruit Experimental Base	953/988 (96%)
C11	DSM 3922	771/774 (99%)	C-ZJB-12-65 (KC354683)source: soil from the orchard in Liujiabao village	771/773 (99%)
C12	DSM 3922	927/950 (98%)	C-ZJB-12-44 (KC354669)source: fruit from the orchard in Changdong village	925/945 (98%)
C13	DSM 3922	813/814 (99%)	FB40 (KF880723)source: debris from factory floor	813/814 (99%)
C14	DSM 3922	878/879 (99%)	FB40 (KF880723)source: debris from factory floor	878/879 (99%)
C15	ATCC 49025	708/725 (98%)	UFRRJAT1 (KC783431.1)source: orange juice	714/725(98%)
C16	ATCC 49025	902/904 (99%)	XC-6 (KJ158157)source: maize juice	902/904(99%)
C17	DSM 3922	896/914 (98%)	C-ZJB-12-12 (KC193187)source: shop environment (walls)	903/921 (98%)
C18	DSM 3922	945/974 (97%)	C-ZJB-12-10 (KC193186)source: kiwi fruits after washing	950/978 (97%)
C19	ATCC 49025	840/851(99%)	XC-6 (KJ158157)source: maize juice	845/855 (99%)
C20	DSM 3922	882/884 (99%)	C-ZJB-12-10 (KC193186)source: kiwi fruits after washing	884/885 (99%)
C21	DSM 3922	800/803 (99%)	C-ZJB-12-64 (KC354682)source: soil from the orchard in Liujiabao village	801/803 (99%)
C22	DSM3922	885/896 (99%)	C-ZJB-12-44 (KC354669)source: fruit from the orchard in Changdong village	886/894 (99%)
C23	DSM3922	882/889 (99%)	C-ZJB-12-17 (KC193190)source: shop environment (raw material bins)	883/887 (99%)
C24	ATCC 49025	1404/1407 (99%)	XC-6 (KJ158157)source: maize juice	1399/1400 (99%)
C25	ATCC 49025	717/721 (99%)	XC-6 (KJ158157)source: maize juice	717/721 (99%)
CB-1	ATCC 49025	1392/1407 (99%)	CB-1 (KP144333)source: pear juice	/
CB-2	ATCC 49025	878/879 (99%)	XC-6 (KJ158157)source: maize juice	878/879 (99%)

*Identification should be confirmed

**Fig 3:**
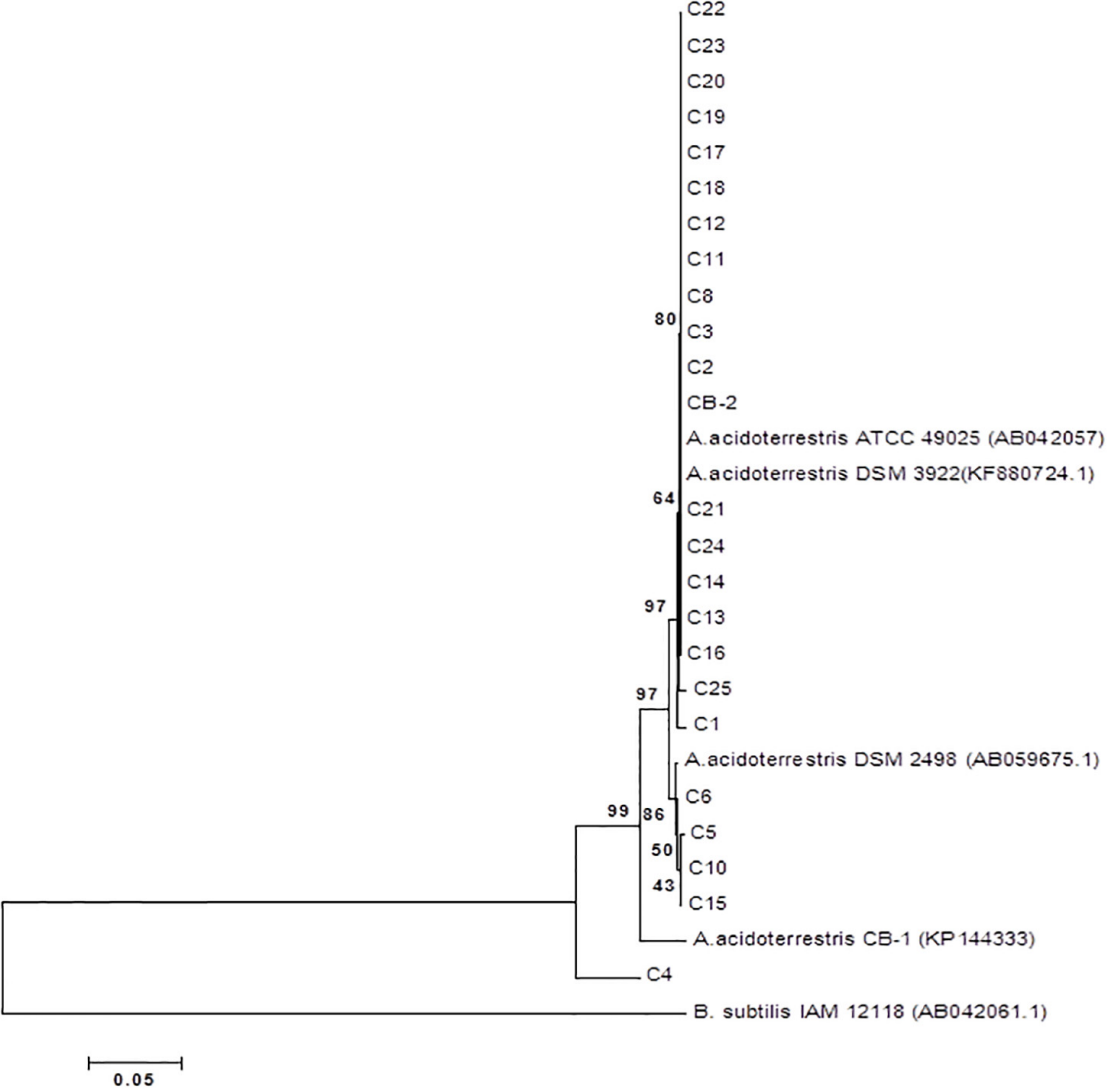
Evolutionary relationships of taxa. (The evolutionary history was inferred using the Neighbor-Joining method. The optimal tree with the sum of branch length = 0.79567517 is shown. The percentage of replicate trees in which the associated taxa clustered together in the bootstrap test (1000 replicates) are shown next to the branches. The tree is drawn to scale, with branch lengths in the same units as those of the evolutionary distances used to infer the phylogenetic tree. The evolutionary distances were computed using the p-distance method and are in the units of the number of base differences per site. The analysis involved 29 nucleotide sequences. All positions containing gaps and missing data were eliminated. There were a total of 573 positions in the final dataset. Evolutionary analyses were conducted in MEGA6.).

The acquired sequences were aligned to evidence nucleotide differences between isolates and reference strains. Sequences analyses revealed the presence of Single Nucleotide Polymorphisms (SNP) that weigh on the phylogenetic tree (Fig 3). Examining of chronometric molecules, such as 16S rRNA, indicates the tendency of organisms to change on a global level and points out on the highly variable regions of 16S rRNA that exhibit high degrees of sequence divergence in even closely related organisms [32]. Results showed that isolates were divided into three blocks, the first block included 12 strains which closest relative is *A*. *acidoterrestris* 49025, the second block incorporated 7 isolates put between reference strains DSM3922^T^ and DSM2498 and finally the third block was represented by 4 strains flanked by reference strain DSM2498 and strain CB-1. C4 strain was not included together with the others since its taxonomic proximity with *B*. *subtilis* IAM 12118 ^T^ used as an outgroup.

Analyzing these blocks in nucleotide database at NCBI website, a curious distribution was observed: the first block, composed of 12 isolates, is divided in 7 isolates (C22-C11 with the exception of C19) that share a great sequence homology with various strains identified by Zhang et colleagues and overall named CZB-12 [33] and 4 isolates (C8-CB-2) that aligned with strain XC-6 (accession number: KJ158157) and strain FB40 (accession number: KF880723) (Table 3). Strain CZB-12 was isolated from the whole production line of kiwi products in China whereas strains XC-6 and FB-40 were identified in maize juice and in the debris from the factory floor respectively. The DNA sequences of the second block, composed of 7 isolates (C21-C1), matched with strains XC-6 (accession number: KJ158157) and FB40 (accession number: KF880723) (Table 3) Finally the third block was formed by 4 isolates (C6-C15) that share a good percentage of identity with strains CBZ-12 [33] and strain UFRRJAT1 [34] found in orange juice (Table 3). This distribution is intriguing since it is possible to speculate that soil isolates were clustered in effectively soil species and isolates that are moving from soil to other possible growing source characterized by parameters, like pH, that could strong influence bacterial survive. It is obvious that this assumption is a mere speculation; nevertheless, it is known that evolving organisms typically share relatively low levels of DNA-DNA homology with other members of the same group and that organisms show the phenotype that may be relevant to some environmental or physiological stress [2]. As reported above, the strains showed variable phenotypic traits, thus these results point out that bacterial species could exist as a genomovar (DNA group within the same species, i.e. strains showing a 70% DNA homology, with different phylogenetic traits but phenotypically indistinguishable) that cover different phenospecies dependent from environmental and physiological stimuli received. The concept of genomovar has been extensively used of many species usually found on soil (*Pseudomonas stutzeri*, *Pseuodmonas cichorii*, *Bacillus licheniformis*, *Burkholderia cepacia*) [35–38] and could be related to genome plasticity, ability to survive and grow in different ecological niche and show only useful phenotypic traits.

Regarding CB-1, its collocation in the phylogenetic tree is relatively distant from *Alicyclobacillus* type strains nevertheless its biochemical profile is indistinguishable from strain CB-2. Genotypically, type strains for many species may not accurately reflect the entire genomic composition of the species; moreover it is possible to hypothesize that CB-1 is a fast-clock organism that typical has a tendency to change on a global level in chronometric molecules, such as 16S rRNA. An interesting trait of population biology is the so-called “molecular clock”; Zuckerkandl and Pauling [39] first described this idea. They plotted the numbers of amino acid differences between the cytochrome c molecules of a number of organisms against the evolution time; thus, they suggested that molecules could turn over in a fairly constant or “clock-like” manner. There are several basic points for this theory; the most important one is that the sequence of a given gene evolves at a constant rate as long as its biological function remains unchanged [40]. Several violations to this generalized statement have been reported, mainly due to horizontal gene transfer in prokaryotes [40]. Nevertheless, the concept of molecular clock is a useful tool to predict expected and unexpected mutations and pinpoint some possible alternative pathways in the evolutionary history. The phylogenetic clustering of the strain CB-1 suggests the beginning of an alternative pathway in alicyclobacilli evolution, mainly for juice strains.

A genotypic indication of a fast-clock organism is an elevated rate of change in more conserved regions compared with the rate of change in the more variable regions of the same molecule [2]. On the other hand, the phenotypic traits are identical to those of CB-2 strain that, like CB-1, was isolated from a spoilage incident in pear juice. It confirms that ecological niche is determinant for genomic activation independently from evolutionary state.

**Fig 4:**
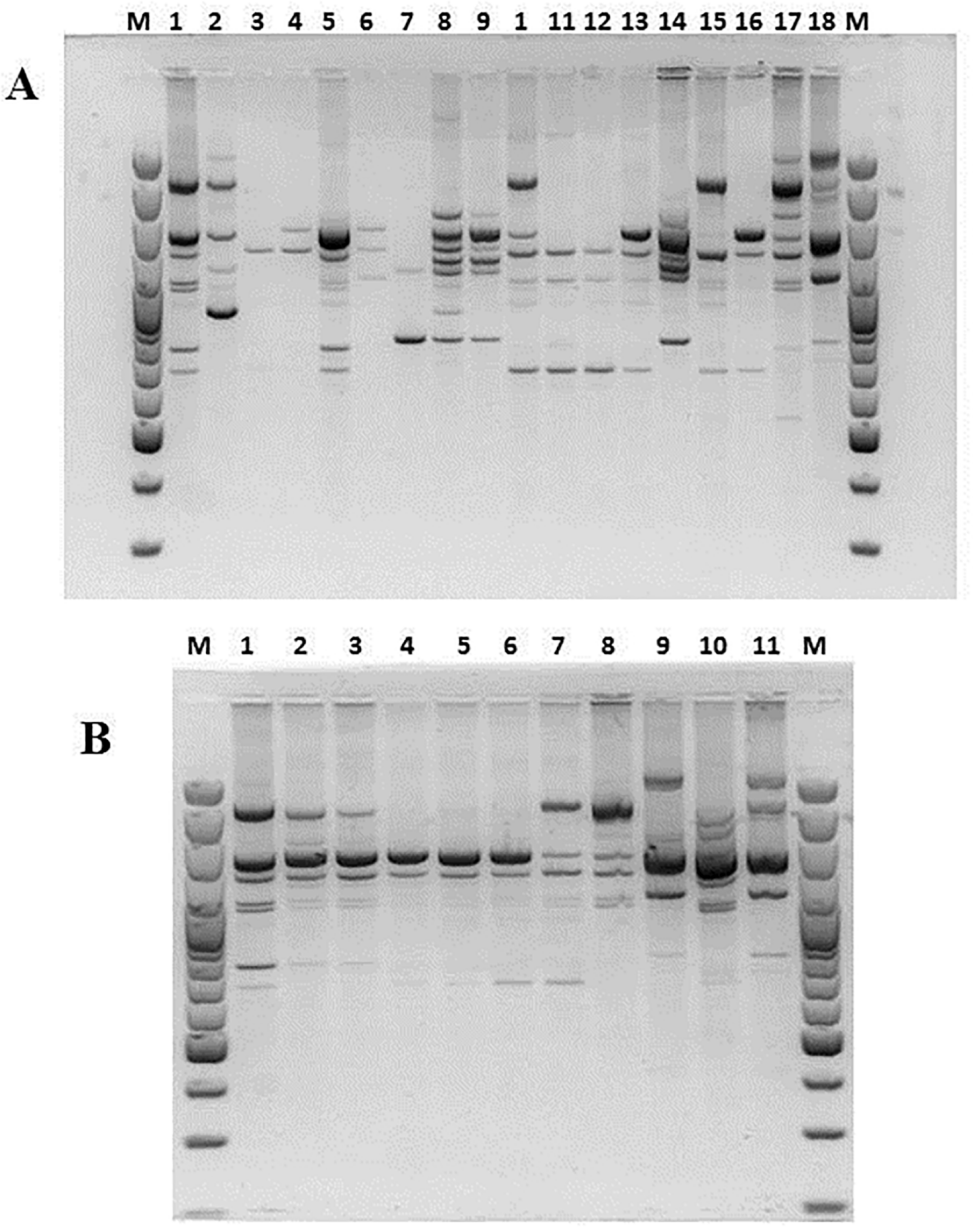
RAPD PCR products obtained amplifying chromosomal DNA of studied *A*. *acidoterrestris* strains as the template, primer BA-10 was used. Section **A**: Lanes 1 and 18: *A*. *acidoterrestris* DSM3922^T^ and DSM2498. Lane 2: C1, lane 3: C2, lane 4: C3, lane 5: C4, lane 6; C5, lane 7: C6, lane 8: C8, lane 9: C10, lane 10: C11, lane 11: C12, lane 12: C13, lane 13: C14, lane 15: C15, lane 16: C17, lane 17: C18. Lanes M: size marker: O’GeneRuler 100 bp DNA Ladder (Thermo Fisher Scientific Inc. MA USA). Section **B**: Lanes 1 and 11: *A*. *acidoterrestris* DSM3922^T^ and DSM2498. Lane 2: C19, lane 3: C20, lane 4: C21, lane 5: C22, lane 6; C23, lane 7: C24, lane 8: C25, lane 9: CB-1, lane 10: CB-2. Lanes M: size marker: O’GeneRuler 100 bp DNA Ladder (Thermo Fisher Scientific Inc. MA USA).

**Fig 5:**
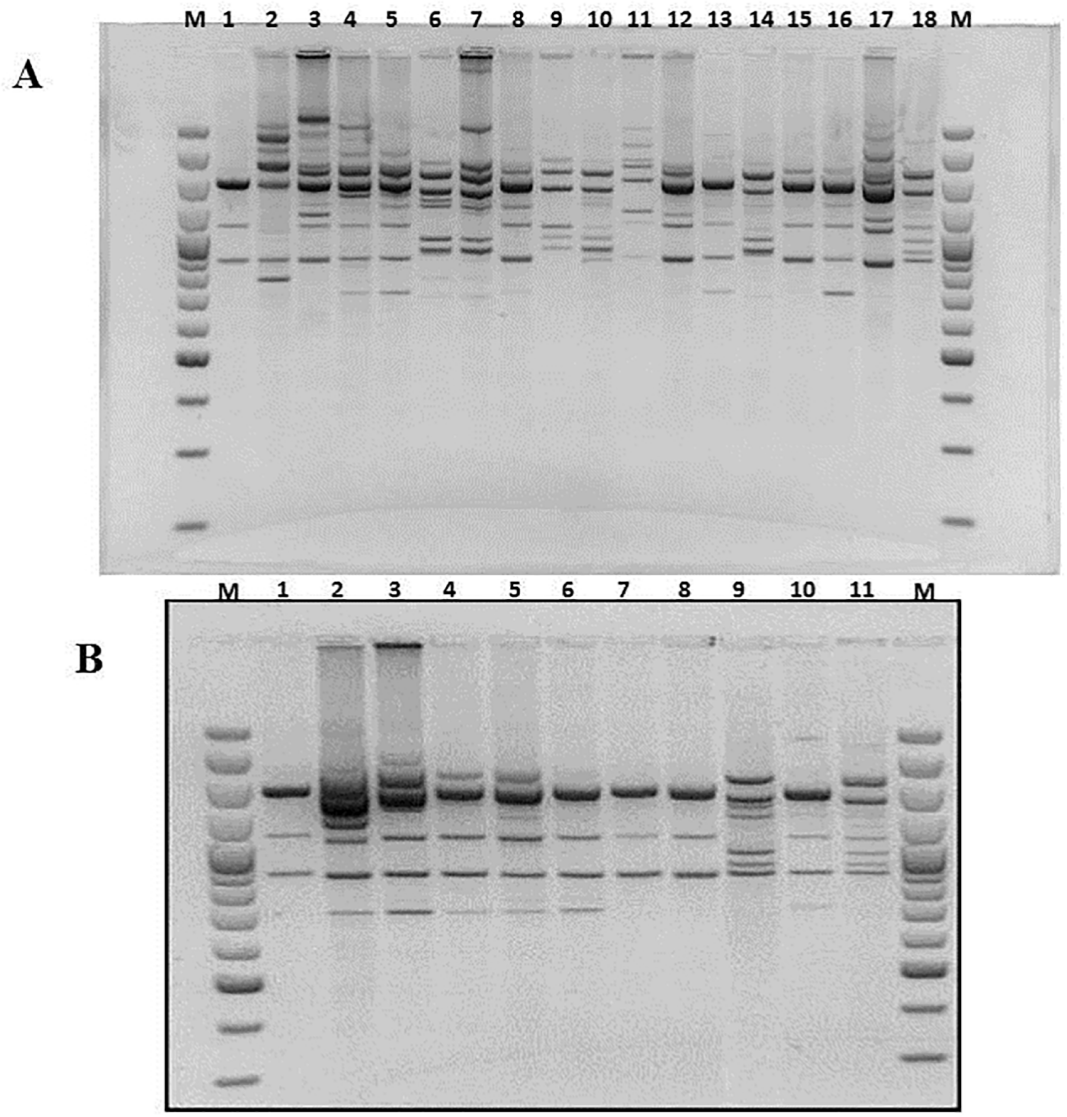
RAPD PCR products obtained amplifying chromosomal DNA of studied *A*. *acidoterrestris* strains as the template, primer F-64 was used.

**Fig 6:**
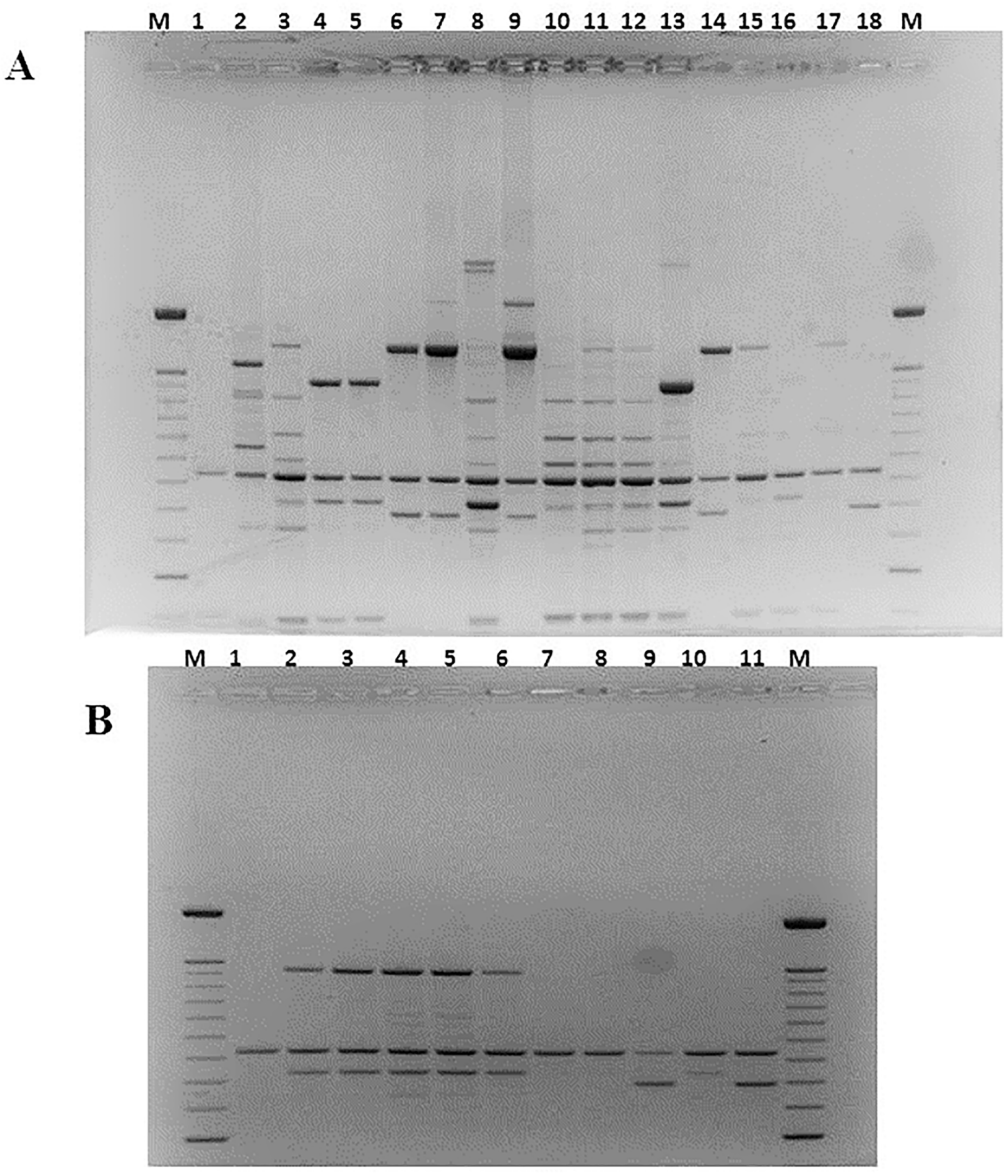
RAPD PCR products obtained amplifying chromosomal DNA of studied *A*. *acidoterrestris* strains as the template, primer F-61 was used.

In the last decade, RAPD PCR technique has been one of the most commonly used molecular techniques to develop DNA markers. RAPD PCR has found a wide range of applications in gene mapping, population genetics, molecular evolutionary genetics, plant and animal breeding [41]. In this study, Yamazaki protocol was employed to generate species-specific banding patterns in order to group strains in clusters [19]. RAPD tests were repeated different times and only the best profile acquired for every studied strain was loaded on the ultimate gel. The results evidenced a great variability in banding patterns and, although it was not possible to obtain genotypically well-distinguished groups, it was feasible to appreciate genetic similarity between strains C19 and C20, strains C21 and C22 and finally between strains C24 and C25 (Figs 4–6). None of strains recovered in the soil presents correspondence with the adopted reference strains.

Regarding CB-2 and CB-1, only CB-1 shared great genetic match with *A*. *acidoterrestris* DSM2498, the considerable concern to fruit juice producers [1–2]. This result suggests that the nature of the growth medium could influence genetic characteristics, moreover, it enables to hypothesize that soil *Alicyclobacillus* could spread into different juice production lines. For instance, CB-1 was first isolated in spoiled pear juice, nevertheless, its surviving and thermal resistance was also assessed in tomato juice causing its spoilage [5].

Used RAPD primers anneal on DNA regions corresponding to molecular chaperones (DnaJ and DnaK), pivotal enzyme (gyraseB) and both subunits of bacterial ribosomes (23S,16S); therefore, RAPD PCR could trace the pathway of strains from soil to juices evidencing that soil-borne strains could evolve to survive under acidity or high temperature conditions. Moreover, using primers F64 and F61, more bands, than primer Ba-10, were generated; it might be caused by the relatively higher affinity of these primers to the genomic DNA of *Alicyclobacillus*. It allowed revealing a genotypic diversity for examined isolates, which indicate the presence of some possible subspecies among them. In fact, as typical in bacterial taxonomy, in order to type a strain, it is essential to gather phenotypic traits and genotypic characteristics because of a set of phenotypes do not accurately reflect the genotypic relationships.

## Conclusion

The investigation of a microbial community entails a combination of metagenomic and classic culture-dependent approaches to expand our knowledge about *Alicyclobacillus* and to look for new subspecies.

Data of soil-borne strains pinpointed that they could be divided into three blocks, represented by soil strains and by strains moving from soil to other niches. In this context, phenotyping and genotyping did not group the strains in the same way and many strains phylogenetically different showed the same phenotypic trend, thus suggesting that *A*. *acidoterrestris* could exist as a genomovar. In addition, the strain CB-1 was distant from other alicyclobacilli, although it possessed the same traits than the other isolates from juice (CB-2); therefore, it is probably a fast-clock organism or the beginning on an alternative pathway in alicyclobacilli evolution.

Finally, the phenotypic traits (pH, temperature, growth with low amount of oxygen) were generally similar to that recovered for *Alicyclobacillus* spp., with some differences (e.g. in salt resistance).
